# Recovery as an intersubjective process: an autoethnography of experiential knowledge in mental health

**DOI:** 10.3389/fpsyt.2026.1851490

**Published:** 2026-07-30

**Authors:** Hideki Muramatsu

**Affiliations:** Graduate School of Arts and Sciences, The Open University of Japan, Chiba, Japan

**Keywords:** autoethnography, expert-by-experience, experiential knowledge, personal recovery, intersubjectivity, social construction

## Abstract

**Objective:**

This study explores how personal recovery is socially constituted in everyday life, providing a novel perspective through the experiential knowledge of an expert-by-experience (EBE) explored using autoethnography. It aims to contribute to the advancement of recovery frameworks by examining the intersubjective dynamics of recovery.

**Methods:**

The data comprised a personal diary maintained over 85 days, beginning April 1, 2024. Inductive thematic analysis was conducted on 2,142 textual units to identify recurring patterns and themes. Analytic rigor was supported through iterative coding, repeated engagement with the data, and reflexive analysis grounded in established autoethnographic principles.

**Results:**

Four overarching themes emerged: (1) interpersonal relationships during recovery; (2) recovery process sensations; (3) environmental factors influencing recovery; and (4) cognition of the recovery process. A key finding is that recovery experiences are deeply relational and socially embedded, shaped by both supportive and challenging interpersonal dynamics as well as environmental constraints. Notably, recovery trajectories frequently include “flat periods” and emotional ambivalence, where positive and negative experiences coexist simultaneously instead of following a simple linear progression.

**Conclusion:**

These findings highlight recovery as a non-linear, intersubjective, and socially constituted process. The study contributes to the conceptual development of recovery by emphasizing the coexistence of ambivalent experiences and the significance of relational and contextual dynamics. For mental health nursing practices, this study suggests the importance of mental health professionals respecting individuals with mental illness’ “flat periods” not merely as stagnation but as part of a complex, socially structured recovery and supporting value-based activities that allow individuals to transcend the salience of their illness even during these periods.

## 1 Introduction

Personal recovery in mental health has become a mainstream and bio-psycho-social approach (1). A positive understanding of mental health recovery is imperative for nursing practice to cultivate empowerment and hope among individuals receiving nursing care (2).

Mental health recovery is a complex concept of multiple interrelated processes and outcomes, supported by access to personal and social resources, referred to as recovery capital (1, 3). Although personal recovery is unique, it has multiple definitions (4) and is a way of living a satisfying, hopeful, and contributing life, even with limitations caused by illness. Recovery involves the development of new meaning and purpose in one’s life as one grows beyond the catastrophic effects of mental illness (5). The CHIME framework for personal recovery in mental health comprises five components: connectedness, hope and optimism, identity, meaning in life, and empowerment (6).

Although the CHIME framework includes connectedness, the individualistic perspective of the Cartesian dualistic worldview downplays relationally oriented views of recovery (1). Recovery is a social process facilitated by relationships (7), social support (8, 9), caregivers (10), informal caregivers (11), family (9, 12), and nurse-patient interaction (13). Previous studies highlight the role of social factors, such as support in employment and housing and community integration, and connectedness, including work relationships, social networks, and perceived support in personal recovery (14, 15). The trajectory toward recovery also highlights the importance of a trusting alliance between nurses or other professionals and people with mental illness (16). Price-Robertson et al. (1) proposed the concept of relational recovery.

In my previous work (17), I proposed the concept of a *socially constitutive process*, which interprets recovery as an intersubjective experience shaped by social factors. It introduces social constructionism (18). In this study, social constructionism is understood as the process through which meanings of recovery are co-constructed through social interaction and embedded in everyday contexts. The international trend emphasizes that recovery should not be discussed solely in terms of individual factors, but rather within the context of complex interactions with environmental and social factors. Despite growing recognition of the social dimensions of recovery, research on how recovery unfolds in everyday life from the first-person experiential perspective of expert-by-experience (EBE) is limited (19–22). It remains conceptually underdeveloped and empirically underexplored. Further consideration is needed regarding the everyday unfolding of the recovery process and the meaning-making perspective in intersubjective dynamics.

Recovery is a long-term process (5) or a lifelong journey (23) involving the struggle to rediscover one’s identity and find new meaning and purpose in life (24). Mental health recovery narratives reflect the recovery process and comprise four trajectory types (25). Previous research identifies several mental health recovery narrative trajectories, such as upward, horizontal, interrupted, and up-and-down patterns, illustrating that recovery is often nonlinear and may involve periods of progress, stagnation, or crisis (25–27).

However, studies suggest that the concept of recovery is sometimes poorly understood in clinical practice. Qualitative research shows that nursing students may lack a clear and thorough understanding of recovery concepts [(28) p. 392]. The concepts of recovery and recovery processes are complex and diverse, involving the interplay of individual and social factors, and research in this area is still developing (29). Existing recovery trajectory frameworks often describe recovery patterns and do not fully capture their subjective experiential meaning in everyday life.

This study advances the literature by examining how recovery is socially constituted in everyday life from an experiential perspective. It aims to explore personal recovery processes from an EBE perspective using autoethnography to provide conceptual insights into recovery in mental health nursing.

This study builds on an autoethnographic study. Autoethnography is a research method that uses personal experience to describe and interpret cultural texts, experiences, beliefs, and practices (30). It is considered effective for re-examining cultural structures and generating new concepts from an insider perspective. It differs from the positivist insider positions, which generate academic knowledge from personal subjectivity, explain tacit knowledge, and improve practice (31). Llewellyn-Beardsley et al. (25) conducted a systematic review of 629 first-person lived experience accounts as a valuable source of scientific information. Knowledge generated from the first-person subjectivity of people with mental disorders and EBEs can generate discussions to revitalize mental health nursing (20–22, 32).

Knowledge of recovery is informed by important insights gained from the personal and subjective stories of people who have experienced psychological distress (25, 32). First-person narratives of mental anguish present new meaning-forming academic insights. For example, some individuals write their narratives during or after recovery to educate others about mental illness, refute stigmas and stereotypes, share useful treatment options, and present their theories about mental illness (26).

As part of this study, I regularly wrote journals providing iterative descriptions of my recursivity (33). This study uses this experiential knowledge to propose progressive ideas regarding recovery in mental health nursing. I present novel insights into my ongoing recovery process from a socially constitutive and intersubjective perspective. Given the complexity of social involvement and the importance of quality in social interactions (14), research focusing on the quality of individuality from an EBE perspective is considered academically valuable.

## 2 Materials and methods

This study employed autoethnography. Although this study proposes a conceptual framework, its purpose is not statistical generalization but the generation of theoretical insights grounded in lived experience (20–22). Autoethnography encompasses multiple methodological styles and approaches (34, 35).

### 2.1 Data

The primary dataset for this study was a diary over 85 days beginning April 1, 2024. My first-person experiential accounts were documented. To continuously collect qualitative data, I recorded short, free-form comments about recovery. The diary was originally written in Japanese. To retain the meaning of the original experiential descriptions, the Japanese text was first segmented into textual units for the descriptive mapping of the data. These units were then translated into English and professionally edited by an English-language editing service. The number of textual units was only used to describe the analytic process, not to indicate the experiential or theoretical significance of each theme.

I attempted to reduce retrospective bias by documenting experiences close to the time they occurred (36). The positionality of the researcher was explicitly stated to provide methodological transparency. Additionally, the diary method (34, 37) has ecological validity (38). It further confirms that my recovery occurs in everyday life and allows me to capture information on contextual and situational factors (38).

Reflexivity and positionality were emphasized, consistent with discussions in autoethnographic methodology (39). Therefore, affiliations and potential self-disclosure bias are presented below.

I have experienced chronic crises with severe depression, frequent panic attacks, derangement of the ego, and cognitive dysfunction (40). I have a master’s degree in child studies and am a Certified Psychologist accredited by the Japanese Psychological Association.

I am an EBE who lives with chronic psychological distress. I have sustained exposure to stressful environments and personal traits (hypersensitivity, fatigability, and anxiety) associated with mental health difficulties (41). I experienced a recovery process facilitated by a relationship of mutual care and reciprocal empowerment with my caregiver (40). After a major crisis, I used aspects of my recovery process to consciously create a reversed power-dynamic relationship in which I, as a caregiver (a different form of caregiving), cared for my mother, fostering my independence, autonomy, and empowerment (40 p. 2). This process led to the progressive construction of intersubjective experiential knowledge through interactions with family, university faculty, mental health professionals, peers, and people with a diagnosis of a mental disorder.

This study met complete member researcher (CMR) status requirements and aimed for ecological validity, consistent with a previous autoethnographic study (41).

The diary entries were analyzed using inductive thematic analysis to identify recurring themes in the recovery process.

### 2.2 Research procedure

This study used inductive thematic analysis to identify patterns and generate themes from the qualitative data (42), chosen for its flexibility and applicability to a wide range of philosophical positions in qualitative research. Thematic analysis allows systematic identification of patterns in qualitative data while maintaining flexibility across different qualitative paradigms (42).

The data were analyzed using inductive thematic analysis (42). The process followed six iterative phases: (1) familiarizing myself with the data through repeated readings; (2) generating initial codes using Microsoft Excel; (3) searching for subthemes; (4) reviewing and refining themes; (5) defining and naming themes; and (6) producing the final report. Each textual unit represented a short segment of free-form diary description that expressed a distinct experiential meaning related to recovery. Initial codes were generated from these units and then compared across the dataset. Codes with similar concrete content and experiential meaning were grouped into subthemes. Broader themes were developed through an iterative movement between the subthemes, original diary excerpts, and the study’s analytic focus on social interactions, care relationships, and empowerment within the recovery process. The consistency between textual units, codes, subthemes, and themes was reviewed repeatedly throughout the analytic process. To enhance analytic rigor, reflexive engagement with the data was maintained throughout, and coding decisions were continuously revisited to ensure consistency and depth of interpretation.

Although no formal external supervision was employed, I attempted to maintain a “helicopter view” of the analysis by moving iteratively between diary excerpts, emerging codes, subthemes, and relevant recovery literature; I also continually examined whether my interpretations were shaped by immediate emotional impressions.

In this reflexive process, I was aware of the dual influence of my positionality as both a certified psychologist accredited by the Japanese Psychological Association and an EBE researcher. As a certified psychologist, I needed to be cautious not to impose theoretical or psychological interpretations too quickly on my diary entries. At the same time, as an EBE, I needed to be cautious not to over-interpret emotionally intense experiences solely from within my immediate subjective feelings. I therefore treated my dual positionality as both a potential source of bias and a methodological resource. My experiential position enabled me to generate knowledge from a partial cultural and relational structure of lived experience, while my psychological training helped me revisit, compare, and mature these interpretations through repeated reading, engagement with recovery literature, and reflexive analysis.

### 2.3 Ethical consideration

Autoethnographic research sometimes employs narrative reconstruction to protect the identity of individuals described in the narrative by modifying or omitting potentially identifiable details. The qualitative data used in this study were anonymized. Member checking was conducted with family members involved in the narrative. The only individuals who might be indirectly identifiable in this narrative were family members. In addition, potentially sensitive and concrete descriptions concerning family members were carefully reviewed from an academic and relational ethics perspective and removed or revised to minimize the risk of identification, misrepresentation, or harm. Family members reviewed relevant sections of the manuscript to ensure that the descriptions were accurate and suitable for publication. Formal ethical approval was not required, as the study was based on the author’s personal experiences and did not involve identifiable external participants, consistent with previous autoethnographic research (21, 22). Relational ethics were considered throughout the research process.

## 3 Results and discussion

Inductive thematic analysis generated four overarching themes and eleven subthemes, revealing the intersubjective and context-dependent nature of the recovery process across 2,142 textual units of qualitative data (**Table 1**).

**Table 1:** Descriptive overview of themes and subthemes developed through reflexive thematic analysis of 2,142 textual units.

Theme	n(%)	Subtheme	n(%)	Specific content
Interpersonal relationships during recovery	754(35.2)	Negative relationships	514(24.0)	close relationships sometimes made the recovery process feel like being in a valley.
		Positive relationships	178(8.3)	others who partially run alongside us in the recovery process had a positive impact on my recovery.
		Other relationships	62(2.9)	some people can share the Umwelt of the recovery process and others cannot.
Recovery Process Sensation	737(34.4)	Stagnant valley floor	267(12.5)	On average, there is a sense of being in “downs”.
		Flat process	187(8.7)	This does not seem to have many ups or downs. Surprisingly, it appears flat and meandering.
		Ambivalence	134(6.3)	In the recovery process, positive and negative elements may exist simultaneously and in parallel.
		Other sense of process	149(7.0)	The sense of recovery process differs between middle age and pre-adolescence.
Environmental Factors	445(20.8)	Transcendence	378(17.6)	Experiences of accepting powerlessness in the face of factors beyond individual control.
		Other environmental factors	67(3.1)	the past process of suffering haunts the recovery process.
Cognition of the recovery process	206(9.6)	Autonomy	128(6.0)	when I was strongly committed to something, the concept of ‘ups or downs’ itself disappeared from my thinking
		Cognition	78(3.6)	changing the quality of the recovery process by transforming the cognition of the recovery process

n indicates the number of textual units coded under each theme or subtheme. These counts are presented only to provide a descriptive overview of the coding process. They were not used to determine the experiential, phenomenological, or theoretical significance of themes; thematic significance was interpreted through reflexive engagement with the diary material and the overall recovery narrative.

The discussion is presented in a semi-structured format (43) following the principles of autoethnography. Interpretation of findings was guided by reflexive analysis, with theoretical insights developed iteratively during the research process (44). Since the diary entries consisted of short free-form comments, some excerpts are presented as composite translated excerpts. Related short entries were combined and lightly edited for readability while preserving the original meaning and analytic context. These entries were written during ordinary periods of living with chronic distress, rather than during a single dramatic event. They illustrate how the meaning of recovery shifted through everyday interactions, relational tensions, and repeated reflections on whether recovery should be understood as upward progress, stagnation, or a more ambiguous process.

### 3.1 Interpersonal relationships during recovery

In the descriptive mapping of the data, challenging interpersonal relationships accounted for 24.0% of the coded textual units. This figure is presented only to describe the coding process and was not used to determine the experiential or theoretical significance of the theme. The interpretive significance of this theme lies in how it illustrates the relational and intersubjective nature of recovery, consistent with previous research that emphasizes recovery as a relational process (1, 45).

“Close relationships sometimes made the recovery process feel like being in a valley. Because of my close contact with the person, the influence I had on them, for better or for worse, was significant. It was precisely because of their intimate relationship that it contained an ambiguity that made it both understandable and incomprehensible.” (Critical reflection).

The theme “Challenging interpersonal relationships” was identified in 514 instances (24.0%) out of 2,142 textual units. Family is the most salient interpersonal context for many people (1, 9, 12).

“People involved in my recovery process influenced my own ‘mountains’ and ‘valleys.’ At times, not only my direct relationships, but also relationships among others around me affected my recovery process. What felt important was the sense that I was not walking through the recovery process alone.”

“I felt a sense of emptiness when I had to remain in long-term relationships with people whose direction in life differed from mine. An isolated recovery process felt strongly negative. It was particularly painful when the interpersonal relationships affecting my recovery process could not easily be repaired.”

Intimate interpersonal relationships vary across individuals, but relationships with family members within the same household may often represent a particularly influential relational context. Families are commonly understood as systems of reciprocal relationships, where the experiences and actions of individual members mutually influence one another (26, 46). In this sense, recovery is not purely individual but emerges through ongoing social interactions. From this perspective, recovery can be understood as an intersubjective process in which the self is shaped through social interactions.

Family relationships are complex and may include both support and tension. They should not be simplified as either wholly supportive or wholly harmful. Even in family environments that are not simply characterized as dysfunctional, difficult interactions, disagreement, and emotional tension may coexist with care, everyday cooperation, celebration of important life events, and support for the author’s future career development, including reviewing and agreeing to the publication of relevant descriptions in this manuscript. This ambivalence illustrates that close relationships may simultaneously constrain and support recovery. Therefore, the purpose of this analysis is not to evaluate or criticize family members, but to examine how close relationships were experienced as part of the author’s recovery process. However, in reality, it is a very complex and ambiguous relationship. However, mitigating the negative aspects is considered important. Evidence on the effectiveness of family psychoeducation and family interventions (47, 48) underscores the importance of family-focused perspectives in mental health practice.

The theme “Positive relationships” was identified in 178 instances (8.3%) out of 2,142 textual units.

“Others who partially walked alongside me in the recovery process became a source of support. Constructive connections with people outside the family made the process feel more positive. When approaches from people around me reduced isolation, my satisfaction with the recovery process increased. Without such connections, the recovery process felt less meaningful.”

“Others who accompany me in the recovery process had a positive impact on my recovery. The journey of life is one in which we share joy, sorrow, and laughter with others. My own life journey, while individual, is also one that is shared through intersubjective interaction with others”. (Critical reflection).

“Others” referred to various individuals involved with oneself, such as family members, peer supporters, and mental health professionals (8, 11, 45, 48). Social interventions (48) and social support (8) by others influence recovery. This excerpt suggests that positive relationships did not function simply as emotional comfort. They also expanded the relational space of recovery by reducing isolation, creating constructive social contact beyond the family, and supporting the author’s sense that the recovery process was shared rather than carried alone.

Furthermore, this supportive influence became particularly important to my development as an EBE. EBEs often face challenges in mental health education (49), including social prejudice. Under these circumstances, several university faculty members (allies) (50) supported me by offering roles to plan and organize mental health-related academic conferences and to serve as a reviewer and breakout session discussant. This support gave me hope (45) and became a catalyst that helped me emerge from a low point during recovery. Recovery involves cultivating positive relationships beyond family (27) and intersubjectively integrating their impact.

Additionally, participation in self-help groups (SHGs), particularly as a facilitator, contributed to my recovery. Through mutual interactions with other participants, both positive and negative experiences were incorporated into my subjective narrative. These interactions illustrate recovery as an intersubjective process, in which the self is continuously shaped through social relationships (17).

These findings suggest that recovery should be understood within relational and family contexts rather than solely as individual psychological change, highlighting it as an intersubjective process.

### 3.2 Recovery process sensation

The subthemes “Stagnant valley floor,” “Flat process,” “Ambivalence,” and “Other sense of process” under the theme “Recovery Process Sensation” capture specific sensations of the recovery process. The subtheme “stagnant valley floor” was identified in 267 instances (12.5%) out of 2,142 textual units.

The subtheme “Flat process” was also identified in 187 instances (8.7%) out of 2,142 textual units. Expressions such as “there is a sense that the recovery process is not characterized by ups and downs” and “it’s a slow, stagnant, flat process” were common.

“I did not feel that my recovery process was simply full of mountains and valleys. Rather, it also felt unexpectedly flat and meandering. I began to feel that evaluating the process as ‘up’ or ‘down’ was not necessarily relevant. There may be a sense of a gentle valley that is also flat. Sometimes an ordinary flat process continues. Even in the case of a mountain, I am not sure whether it is always good to keep going upward.”

My recovery trajectory shifted from non-recovery to a temporary upward phase and later stabilized into a horizontal pattern. This suggests that recovery may not always follow upward or fluctuating trajectories but may instead involve prolonged stability or stagnation. These diary entries show that the “flat” process was not merely an absence of change or a horizontal trajectory. Rather, it reflected a shift in how the recovery process was evaluated. The distinction between “up” and “down” became less central, and flatness was experienced as a way of suspending hierarchical judgments about recovery progress.

The subtheme “Other sense of process” was identified in 149 instances (7.0%) out of 2,142 textual units. Specifics included statements such as, “The sense of recovery process differs between middle age and pre-adolescence.” These findings suggest that the experience of recovery may vary across the life course, as middle age involves distinct social roles and transitions that shape mental health experiences (51).

The subtheme “Ambivalence” was identified in 134 instances (6.3%) out of 2,142 textual units.

“In the recovery process, positive and negative elements may exist simultaneously and in parallel. Life’s journey is a mix of “good moods” and “bad moods” even within a single day. These can cancel each other out, manifest alternately, or exist ambiguously. The recovery process often feels like a very complex concept”. (Critical reflection).

The findings suggest that recovery may involve experiential states that are not fully captured by existing narrative categories, including stagnation, flat trajectories, and emotional ambivalence.In some cases, positive and negative elements cancel each other out, producing flattened experiences. However, in other instances, they coexist in ambivalence. Although previous studies have identified expressions such as encountering the normal ups and downs of life (27, 45), this study suggests that recovery is not necessarily a dualistic concept in which only one positive or negative element prevails. For example, during a period of academic success that brought happiness and a sense of achievement, I simultaneously experienced strong negative emotions related to stigma and challenging interpersonal encounters in mental health contexts (52). This represented a “down” state. In this case, positive and negative experiences did not cancel out; instead, I simultaneously felt happiness alongside the conflicting emotions of anger and sadness. These findings suggest that recovery may involve complex states in which positive and negative elements coexist simultaneously, highlighting the need for more nuanced conceptualizations. This finding suggests that recovery-oriented practice should avoid interpreting positive and negative experiences as mutually exclusive and should instead attend to the ambivalent emotional textures of everyday recovery.

### 3.3 Environmental factors in the recovery process

Environmental factors, including the subthemes “Transcendence” and “Other environmental factors,” were identified in the analysis. Environmental factors included difficulties finding a community to belong to, uncertainty about employment and academic pathways, financial constraints, stigmatization, and weakening social relationships. A trend toward lower strength and quality of social support, networks, and relationships (8) was also identified, alongside limited access to opportunities and enjoyment (45).

The subtheme “(Accepting powerlessness) Transcendence” was identified in 378 instances (17.7%) out of 2,142 textual units. Despite efforts to improve difficult situations, many factors—including developmental traits, family environments, and unexpected life events—remained beyond individual control, often generating a sense of powerlessness during recovery.

Transcendence in this study did not necessarily refer to a religious or spiritual experience. Rather, it described the felt presence of forces beyond individual control that shaped the recovery process. One composite diary excerpt captured this experience: “*There are environmental consequences in my past recovery process that I cannot change by my own effort. My life process seems to be influenced more by the environment than by my own self. There are many things in life that cannot be changed even when I do what I should do. The recovery process is not purely individual; it meanders under the pressure of uncertainty, past time, environment, and forces beyond my control*.

“I believe I did everything I could, taking full responsibility for my actions. However, during the recovery process, I encountered numerous problems that were beyond my control or ability to solve from a biological, psychological, and social perspective. Commit to the process and remain detached from the outcome”. (Critical reflection).

Despite my best efforts, I often experienced an overwhelming sense of powerlessness against forces far beyond my control. These findings highlight the limits of individual agency within broader environmental and social constraints. This interpretation is consistent with sociologically informed recovery research (53), which suggests that socio-structural disadvantage may negatively influence personal recovery as one factor among multiple biological, psychological, relational, and environmental conditions. Thus, recovery difficulties should not be reduced to individual motivation or self-management alone.

For example, from a “biological” perspective, there was the decline in physical function during middle age. From a “psychological” perspective, there was the difficulty in improving interpersonal relationships on a partial basis (41). From a “social” perspective, the significant stigma against researchers was often beyond my control (52). Additionally, individuals with mental illness may experience the life catastrophes described by Anthony (5), compounded by chronic mental health difficulties. Trauma and disaster research suggest that externally imposed events, such as natural disasters, serious accidents, and violence, can negatively affect mental health beyond the individual’s immediate control (54, 55).

In this study, transcendence referred to this experience of overlapping difficulties during recovery, in which biological, psychological, relational, and social burdens accumulated and constrained recovery. This finding complicates the hopeful claim that “there is no limit to recovery.” Although, this statement can be valuable as an aspirational message that resists pessimism, stigma, and deterministic views of mental illness, experiential knowledge suggests that recovery cannot be understood as unlimited solely through individual will, motivation, or self-management. Recovery may be oriented toward possibility and growth, yet it is also lived within biological, psychological, relational, environmental, and social limits. At the same time, recognizing these limits does not mean denying recovery or adopting a fatalistic position. Rather, it highlights the need to address the conditions that restrict recovery through stronger social support (8), more accessible services, family and community support (56), anti-stigma efforts, and environments that enable people to rebuild their lives after adversity (53). In this sense, the present analysis reveals a paradox within clinical and personal recovery discourse: recovery may be imagined as limitless as a hopeful ideal, but in lived experience, the expansion of recovery possibilities depends partly on transforming the social and environmental conditions that constrain them.

### 3.4 Cognition of the recovery process

The theme of process cognition was identified with two subthemes: autonomy and cognition. The theme “Cognition of processes” was identified in 206 instances (9.6%) out of 2,142 textual units. Under the subtheme cognition, the specific content centered on changing the quality of recovery by transforming how the process is cognitively interpreted.

The flat recovery process (FRP) refers to a non-hierarchical interpretation of recovery trajectories in which periods of stagnation or flatness are not considered failure or regression but as meaningful phases within the broader recovery process (41). FRP should not be understood as a horizontal trajectory itself or merely as a subtype of horizontal recovery. Rather, FRP refers to a subjective and value-based reframing of how recovery trajectories are experienced. From the perspective of FRP, different recovery processes are not evaluated according to hierarchical assumptions of progress, success, or failure. Instead, “flat” refers to the universal equality of human value, emphasizing that a person’s life should not be ranked as superior or inferior according to whether their recovery appears upward, downward, stagnant, or meandering. In this sense, FRP is less a distinct trajectory type than a conceptual lens for interpreting recovery experiences without reducing them to vertical judgments of improvement or decline. The FRP perspective aligns with person-centered care by recognizing individuals with mental illness as whole persons rather than defining them through deficits (28).

The autoethnographic analysis suggested that the concept of FRP did not substantially change experiences during periods of severe distress. In contrast, during periods of moderate illness and stagnation, the concept helped reduce agitation, self-doubt, and shame. However, this study was a qualitative, personal investigation.

“The concept of FRP was helpful to me in suppressing feelings of stagnation and guilt during the recovery process when I was feeling stable or slightly unwell. However, when I was feeling quite severely unwell, the concept of FRP seemed almost metaphysical”. (Critical reflection).

In recovery, one factor may be the focus of thoughts on “downs” during severe psychosomatic distress. Emotional states may influence how individuals interpret recovery. Future research should explore the conditions under which the FRP concept is effective and when it is not, depending on the intensity of psychosomatic discomfort. Further quantitative research, including fine-grained qualitative research, to enhance understanding and generalizability, is warranted.

The subtheme “Autonomy” was identified in 128 instances (6.0%) out of 2,142 textual units. Both internal distress and external circumstances limited autonomy, consistent with the competence condition described by Bergamin et al. (57). My internal dysfunctional factors have reduced compared with a few years ago (40, 41). However, managing daily stressors alone cannot avert distress, as external factors are less modifiable and personal circumstances do not change the environment, resulting in learned helplessness.

One description encapsulates this complexity: “When I was strongly committed to something, the concept of ups or downs itself disappeared from my thinking.” This observation reflects principles of acceptance and commitment therapy (ACT), emphasizing committed action aligned with personal values (58). In this study, value-based activities refer to personally meaningful activities aligned with my values and identity.

In my case, as an EBE, the emphasis was on performing academic activities, such as writing papers and presenting posters at conferences. This observation reflects principles in ACT, emphasizing committed action aligned with personal values (58). When I focused intensively on academic activities, these value-based actions appeared to reduce the salience of stigma and illness.

This state enabled me to temporarily forget negative perceptions that added to the recovery process. Intensive commitment to my values, rather than intentional awareness of the recovery process itself, served to diminish the salience of mental disorder within my social identity. In my case, it became clear that even in the short term, autonomy was not exercised when I was suffering from chronic physical and mental illnesses. However, sometimes autonomy was exercised by being committed to my values, ​​even when ill; it was a very complex state. These dynamics suggest that single interview studies may capture only fragments of the recovery process, depending on the timing and context of the interview.

## 4 Incremental contribution of this study

This study extends my previous work in several ways. My earlier work on the FRP proposed a non-hierarchical interpretation of recovery trajectories, in which stagnation or flatness is not necessarily regarded as failure or regression (41). However, the present 85-day diary-based autoethnographic analysis adds an experiential examination of conditions under which the FRP concept may or may not be helpful. The findings suggest that FRP may reduce agitation, shame, and self-doubt during periods of relative stability or moderate distress but may become less practically accessible during periods of severe psychosomatic distress. In this sense, the present study contributes by suggesting the state-dependent usefulness of FRP, even though this remains a single-case and temporally situated finding.

This study also extends my previous conceptual work on socially constituted recovery (17). Whereas earlier work proposed recovery as a socially constituted process at a conceptual level, the present analysis shows how this process was experienced in everyday life through relational ambivalence, challenging and supportive interpersonal relationships, and interactions. The findings add diary-based experiential detail to the concept of socially constituted recovery by showing that relational contexts may simultaneously support and constrain recovery.

Finally, the present study adds the concept of transcendence, understood here as accepting powerlessness before biological, psychological, relational, environmental, and social conditions beyond individual control. This dimension was not fully developed in my previous work (17, 41). The present findings suggest that recovery cannot be understood solely through individual agency, motivation, or self-management. Rather, experiential knowledge indicates that recovery may be oriented toward possibility while also being lived within limits that exceed the individual. These contributions clarify how the current study both differs from and extends my prior publications.

## 5 Clinical and nursing implications

The findings have several implications for mental health nursing, peer support, and family support practices. First, the concept of flat periods suggests that nurses and other mental health practitioners should be cautious not to pathologize periods of stagnation or apparent lack of progress. In recovery-oriented practice, such periods may not necessarily indicate relapse, failure, or lack of motivation. Rather, they may represent meaningful phases in which a person is maintaining life, reorganizing values, or living through difficult conditions. It may help practitioners avoid focusing only on upward progress or simple good/bad evaluations of recovery.

Second, the findings suggest that interpersonal and family relationships should not be understood through simple binary categories such as supportive versus harmful. Family relationships may involve tension, care, conflict, cooperation, dependence, and support at the same time. For nurses, therapists, peer workers, and family support practitioners, this indicates the need to understand family contexts with greater nuance. Rather than assuming that a family relationship is either a resource or a barrier, practitioners may need to assess how relational contexts simultaneously support and constrain recovery. This may contribute to more sensitive family psychoeducation, family intervention, and relational support practices.

Third, this study illustrates the practical value of experiential knowledge generated by an EBE. The findings suggest that EBE knowledge can contribute not only to recovery theory but also to mental health nursing, peer support, and psychiatric rehabilitation by making visible aspects of recovery that may be difficult to capture through professional observation alone. In particular, the experiential accounts of flat periods, ambivalence, relational recovery, and transcendence may help practitioners reflect on how recovery is lived in everyday contexts. This supports the further inclusion of EBE perspectives in education, practice development, and collaborative research.

## 6 Conclusion

This study provides a novel intersubjective and socially constructed perspective on the recovery process, grounded in experiential knowledge from an EBE using autoethnography. Four themes were identified (1): Interpersonal Relationships during Recovery (2), Recovery Process Sensation, (3) Environmental Factors in the Recovery Process, and (4) Cognition of the Recovery Process.

Recovery requires cultivating positive relationships (27) and intersubjectively integrating their impact. The family is an ambiguous concept encompassing both positive and negative elements. Further research is needed to understand more deeply how safe and secure relationships are fostered within the family system as a whole, rather than focusing solely on individual members through reductionist discussions.

Recovery Process Sensation refers to a specific sensation experienced during recovery. The recovery process is neither systematic nor planned (5). The findings suggest that recovery cannot be fully described as a simple trajectory of ups and downs. Instead, positive and negative experiences may coexist simultaneously, indicating the need for more nuanced conceptualizations.

Environmental factors beyond individual control were also found to influence recovery. These findings highlight recovery as a socially constituted process, in which personal experiences are shaped through social interactions and broader environmental contexts. This perspective may complement existing relational approaches to recovery and contribute to discussions related to the social model.

Regarding the Cognition of the Recovery Process, the results of the individual qualitative analyses revealed differences in the effectiveness of FRP (framework of recovery process) (41) recognition across contexts in reframing subjective distress during periods of stagnation. Also, this study suggests the importance of mental health professionals respecting persons with mental illness’ “flat periods” not merely as stagnation but as part of a complex, socially structured recovery and supporting value-based activities (e.g., work and creative pursuits) that allow individuals to transcend the salience of their illness even during these periods.

This study contributes to recovery literature by providing experiential insights into how recovery unfolds in everyday life. The findings highlight the intersubjective and contextual dynamics of recovery, advancing current recovery frameworks and offering practical and conceptual implications for mental health nursing research and practice. However, the findings should be interpreted in light of the study’s autoethnographic and single-case design, which prioritizes depth of insight over generalizability. Furthermore, because this study focused primarily on my internal recovery processes, future research should examine in greater detail how support from informal caregivers and professionals may facilitate recovery.

## Data Availability

The original contributions presented in the study are included in the article/supplementary material. Further inquiries can be directed to the corresponding author.
